# Interfacial Reactions and Mechanical Properties of Sn–58Bi Solder Joints with Ag Nanoparticles Prepared Using Ultra-Fast Laser Bonding

**DOI:** 10.3390/ma14020335

**Published:** 2021-01-11

**Authors:** Gyuwon Jeong, Dong-Yurl Yu, Seongju Baek, Junghwan Bang, Tae-Ik Lee, Seung-Boo Jung, JungSoo Kim, Yong-Ho Ko

**Affiliations:** 1 Advanced Functional Technology R&D Department, Institute of Industrial Technology (KITECH), 156 Gaetbeol-ro, Yeonsu-gu, Incheon 21999, Korea; gyuwon@kitech.re.kr (G.J.); alpha0987@kitech.re.kr (D.-Y.Y.); back9457@kitech.re.kr (S.B.); nova75@kitech.re.kr (J.B.); tilee@kitech.re.kr (T.-I.L.); 2 School of Advanced Materials Science & Engineering, Sungkyunkwan University, 2066 Seobu-Ro, Jangan-gu, Suwon, Gyeonggo-do 16419, Korea; sbjung@skku.edu; 3 Department of Material Science and Engineering, Korea University, 145 Anam-ro, Seongbuk-gu, Seoul 02841, Korea; 4 Department of Material Science and Engineering, Incheon National University, 119 Academy-ro, Yeonsu-gu, Incheon 22012, Korea; 5 Department of Industrial Technology, University of Science and Technology, 217 Gajeong-ro, Yuseong-gu, Daejeon 34113, Korea

**Keywords:** low-temperature bonding, laser process, Ag nanoparticle (Ag NP), interfacial reaction, intermetallic compounds (IMCs), mechanical property

## Abstract

The effects of Ag nanoparticle (Ag NP) addition on interfacial reaction and mechanical properties of Sn–58Bi solder joints using ultra-fast laser soldering were investigated. Laser-assisted low-temperature bonding was used to solder Sn–58Bi based pastes, with different Ag NP contents, onto organic surface preservative-finished Cu pads of printed circuit boards. The solder joints after laser bonding were examined to determine the effects of Ag NPs on interfacial reactions and intermetallic compounds (IMCs) and high-temperature storage tests performed to investigate its effects on the long-term reliabilities of solder joints. Their mechanical properties were also assessed using shear tests. Although the bonding time of the laser process was shorter than that of a conventional reflow process, Cu–Sn IMCs, such as Cu_6_Sn_5_ and Cu_3_Sn, were well formed at the interface of the solder joint. The addition of Ag NPs also improved the mechanical properties of the solder joints by reducing brittle fracture and suppressing IMC growth. However, excessive addition of Ag NPs degraded the mechanical properties due to coarsened Ag_3_Sn IMCs. Thus, this research predicts that the laser bonding process can be applied to low-temperature bonding to reduce thermal damage and improve the mechanical properties of Sn–58Bi solders, whose microstructure and related mechanical properties can be improved by adding optimal amounts of Ag NPs.

## 1. Introduction

Recently, electronic devices have been used in microelectronic package technologies [1,2,3] with advanced capabilities, such as multi-functions, high densities of input/output, and high capacities. To achieve high performance and miniaturization, various packages are mounted on an electronic substrate. The mechanical properties of bonding joints between packages and printed circuit boards (PCBs) may degrade due to thermal damage and coefficient thermal expansion (CTE) mismatches in the coefficients of thermal expansion during multiple bonding processes. To solve these problems, local low-temperature bonding processes are required.

Prior to the 2000s, Sn–Pb solder alloys were the most commonly used joint materials because of their excellent properties, such as wettability, low melting temperature, and cost competitiveness. However, the use of Pb was banned by the Restriction of Hazardous Substances Directive and Waste Electrical and Electronic Equipment because of its toxicity to humans and the environment [1,2,3,4,5]. Therefore, various kinds of Pb-free Sn-based solder families, such as Sn–Ag, Sn–Cu, Sn–Zn, Sn–Bi, and Sn–In, have been developed to replace the Sn–Pb solder [6,7,8]. Among these, Sn–Ag–Cu solder alloys are widely used as joint bonding materials. However, it is difficult to apply these alloys at low temperatures due to their high melting points (~217 °C) [9,10]. Therefore, Pb-free solders with low melting temperatures, such Sn–Bi and Sn–In, have been used for low-temperature bonding. Sn–In solder alloys have limited application and productivity in the packaging industry owing to high cost [7,9]. Sn–Bi alloys have several advantages, such as high yield strength, creep resistance, and low cost [11]. However, the Sn–Bi solder also has disadvantages such as degradation of mechanical properties and poor reliabilities stemming from the Bi. To overcome these drawbacks, researchers have tried to prevent coarsening and brittle fracture of Bi by adding elements or particles such as Ag, Sb, epoxy, and carbon nanotubes to Sn–58Bi solders [11,12,13,14,15]. In particular, the addition of Ag nanoparticles (Ag NPs) to Sn–58Bi solders is known to affect the microstructures in the bulk and reduce the growth of intermetallic compounds (IMCs) at the interface of the solder joint, which reduces diffusion of Bi and Sn [13].

Soldering, which is a reflow process based on surface mount technology, is the most commonly used process for bonding between some electronic packages and substrates for second level packaging. This is because soldering is capable of being used in mass production at low costs [16]. However, the conventional reflow process is difficult to use for bonding with miniaturized electronic components and may cause thermal damage [17,18,19]. Therefore, the abilities of laser bonding to reduce thermal damage by minimizing fast heating and bonding time has been studied [20]. In this process, applied to miniaturized electronic components, a laser can locally irradiate materials, such as electronic components, solder alloys, and substrates. In addition, a laser beam is rapidly generated by increasing the vibration energies of molecules in the materials in the packages. Thus, electronic components and substrates can be joined by transferring the heat generated in the substrates to bonding joints or solder joints between different components and substrates. This results in the completion of fabrication of second level packages in a much shorter process [21,22,23]. As a result, the laser process has many advantages, such as localized heating, shorter bonding time, and non-contact heating, compared to the conventional reflow soldering process. In addition, if a low-temperature solder like Sn–58Bi is used, it is possible to reduce the thermal damage and deterioration during laser bonding because of the low melting temperature of Sn–58Bi and the localized heating of the laser process. Previous studies [17,22] have shown that the bonding strength from the laser bonding process was higher than that for the conventional reflow process. It was also confirmed that the IMC’s thickness and grain size were smaller for laser bonding compared to those for the conventional reflow process due to the shorter bonding time of the former. However, studies showing improved mechanical properties of Sn–58Bi solder joints, which are currently brittle, are still lacking.

In this research, interfacial reactions and mechanical properties of the Ag NP reinforced Sn–58Bi solder with PCBs of organic surface preservative (OSP)-finished Cu pads were investigated. The joints were formed by using the laser soldering process. To examine the effects of adding different amounts of Ag NPs to the Sn–58Bi solder on the properties of the joints, microstructures in the solder bulk and IMCs produced by reactions at the interface were analyzed. In addition, shear tests were performed on the Ag NP reinforced Sn–58Bi solder at two different speeds to evaluate the mechanical properties of the joints. To analyze long-term reliability, high-temperature storage (HTS) tests were also conducted at three different temperatures. After the HTS tests, the effects of Ag NPs on joint properties, such as microstructures, interfacial reactions, IMC growth, and mechanical properties, were also analyzed and related to different Ag NP contents and different HTS test conditions.

## 2. Materials and Methods

**Figure 1:**
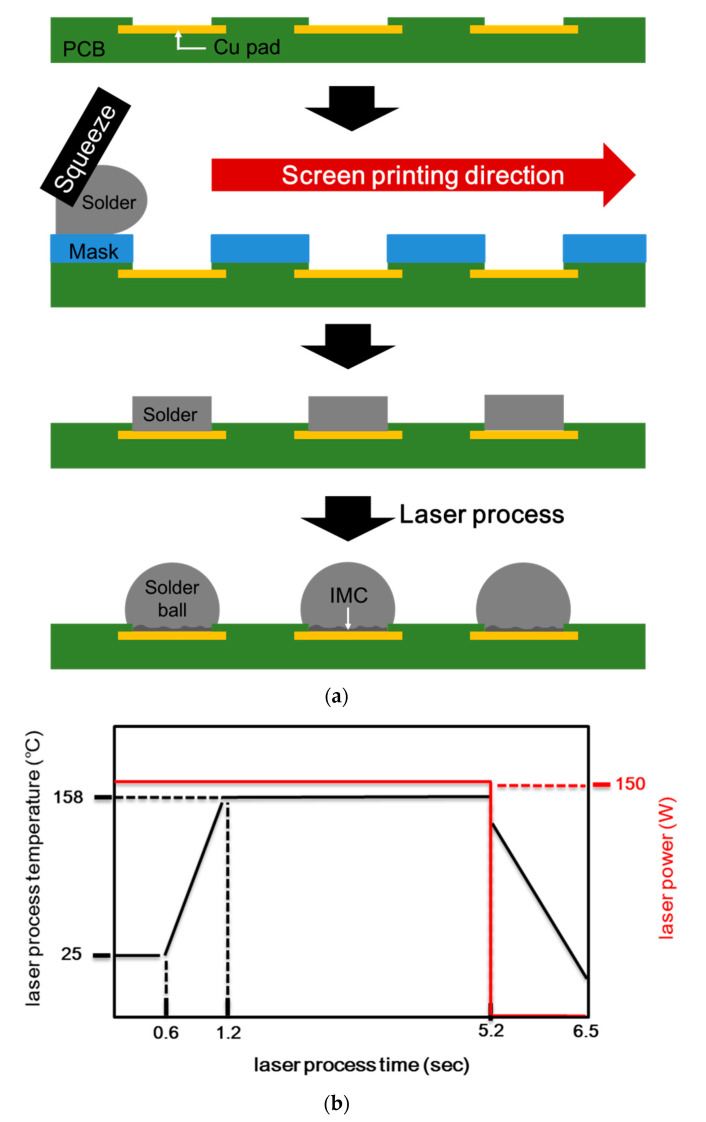
(**a**) Schematic of the fabrication process for test samples and (**b**) profiles for bonding conditions by the laser process.

For this study, Sn–58Bi composite solder pastes with added Ag NPs were fabricated using Sn–58Bi solder powders (20–38 μm; Avention, Incheon, Korea), Ag NPs (20 nm; Avention, Korea), and a low-temperature flux (CVP-520, Alpha Co., Somerset, NJ, USA). The compositions of the solder pastes are given in Table 1. It was weighted on a scale (Practum224-1SKR, Göttingen, Neddersassen, Germany). To make the pastes, the flux was first mixed with Ag NPs to correspond to 0.0 wt.%, 0.5 wt.%, 0.75 wt.%, 1.0 wt.%, and 2.0 wt.% of Ag NPs. Mixing was carried out for 5 min at a speed of 800 revolutions per minute (RPM) under revolution and rotation using a paste mixer (paste mixer; PDM-300V, Daewhatech, Yongin, Korea). The solder powders were then mixed with flux and Ag NPs. The composite solder pastes were printed on OSP-finished Cu pads with a diameter of 300 μm on the flame retardant 4 (FR-4) PCB substrates. A screen-printing method was used with a stencil mask with a thickness of 100 µm and an open size of 760 µm (Figure 1). After the Sn–58Bi Ag NP composite solder paste was printed onto the Cu pads on the PCB, a laser soldering process was performed for 4 s at a peak temperature of 158 °C (laser power of 150 W) using a laser energy source (CW Fiber lasers, IPG Photonics, Oxford, MA, USA). After the laser solder process and the formation of solder joints, flux residues were removed and cleaned using a de-flux solution. The fabrication process and the conditions of the laser soldering process are shown schematically in Figure 1.

To analyze how the joint properties changed with the addition of Ag NPs, scanning electron microscopy (SEM; Inspect F, FEI Co., Hillsboro, OR, USA) and energy-dispersive spectrometry (EDS; superdry, Thermo Noran Co., Waltham, MA, USA) were performed to observe the microstructure, interfacial reaction, and IMC formation at the interface of the solder bulk and solder joints. The mechanical properties of the solder joints were evaluated under two shear test conditions. The shear tests were performed at two shear speeds of 0.1 and 1.0 m/s with a shear height of 50 μm using a high-speed shear tester (Dage 4000 HS; Nordson Co., Aylesbury, Buckinghamshire, UK). In the case of a shear speed of 0.1 m/s, the general shear strength was confirmed, and in the case of a shear speed of 1 m/s, the impact mechanical property was measured. Low- and high-speed shear tests were performed according to the Joint Electron Device and Engineering Council (JEDEC) 22-B117A standards. In addition, the fracture surfaces were investigated after the shear test, and the additional effect of Ag NPs on the mechanical properties was analyzed using SEM and EDS.

HTS tests were conducted to analyze the effect of Ag NPs on the long-term reliabilities of the laser processed solders. The HTS tests were performed for 1000 h at three temperatures: 85, 100, and 115 °C. This environmental reliability test was based on the JEDEC JESD-47 and JESD-A103E standards. After the HTS tests, the effects of the addition of Ag NPs on the microstructures, formation of IMCs, and mechanical properties were investigated. The effects of Ag NP content and the temperature conditions on the thickness and growth of the IMCs were also analyzed during the HTS tests.

## 3. Results and Discussion

**Figure 2:**
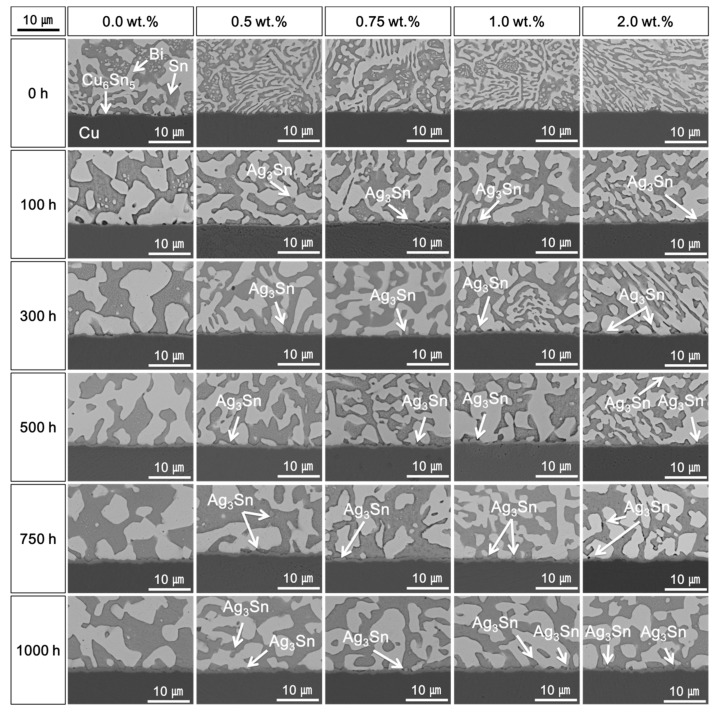
Cross-sectional scanning electron microscopy (SEM) micrographs of the Sn–58Bi Ag NP composite solder joints after high-temperature storage (HTS) tests at 85 °C.

**Figure 3:**
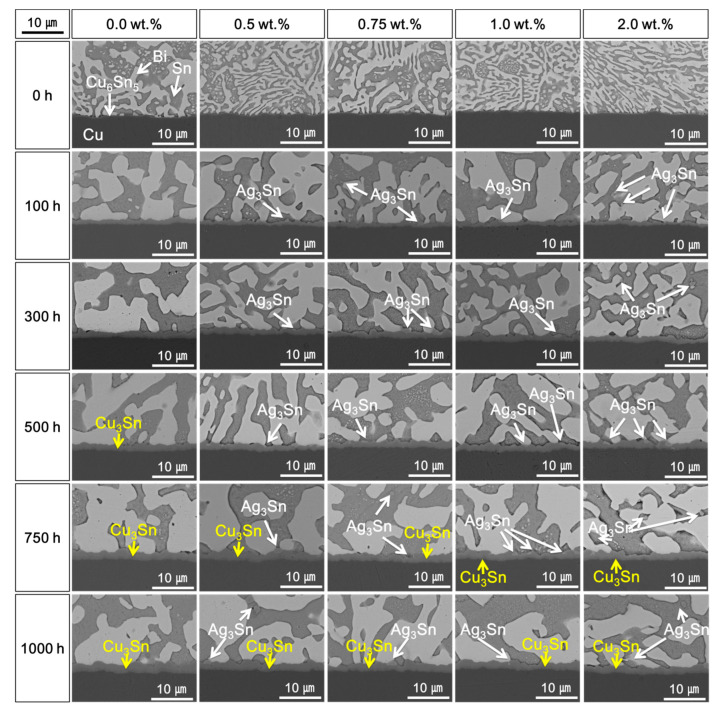
Cross-sectional scanning electron microscopy (SEM) micrographs of the Sn–58Bi Ag NP composite solder joints after high-temperature storage (HTS) tests at 100 °C.

Figure 2 shows cross-sectional SEM micrographs of the solder joints with different Ag NP contents during the HTS test at 85 °C for 1000 h. After reflow, a Cu_6_Sn_5_ IMC layer formed at the interface between the Sn–58Bi solder and the OSP-finished Cu pads regardless of the Ag NP content in the solder pastes. The Sn and Bi elements formed eutectic lamellar structures in the Sn–58Bi solder because of the similar volume percent of Sn and Bi [15,24]. The initial Cu_6_Sn_5_ IMC layer thicknesses did not vary significantly from 0.0 to 2.0 wt.% Ag NPs, with values of 0.25, 0.24, 0.21, 0.20, and 0.22 μm. However, the Cu_6_Sn_5_ IMC layer thicknesses were increased with increasing HTS time. In addition, a plate-type Ag_3_Sn IMC was formed by the addition of Ag NPs after HTS for 100 h. The cross-sectional SEM micrographs of the Sn–58Bi Ag NP composite solder joints after the HTS test at 100 °C for 1000 h are shown in Figure 3. As the HTS time increased, the Cu_6_Sn_5_ IMC layers of the solder joints treated at 100 °C were thicker than those treated at 85 °C. The total Cu–Sn IMC layer thickness decreased with increasing Ag NP content. Island-type Cu_3_Sn IMC was observed at the interface between the Cu_6_Sn_5_ IMC layer and the OSP-finished Cu pad of the Sn–58Bi solder joint without Ag NPs after 500 h under the HTS test. For solders with Ag NP contents above 0.5 wt.%, after 750 h, Cu_3_Sn IMC was observed. The Cu_6_Sn_5_ IMC was then decomposed into Cu_3_Sn IMC and Sn to form Cu_3_Sn IMC at the interface [25,26]. In the case of the Sn–58Bi Ag NP composite solder, it is thought that the formation of Cu_6_Sn_5_ IMC was suppressed by Ag_3_Sn IMC, and the growth of Cu_3_Sn IMC was also suppressed.

**Figure 4:**
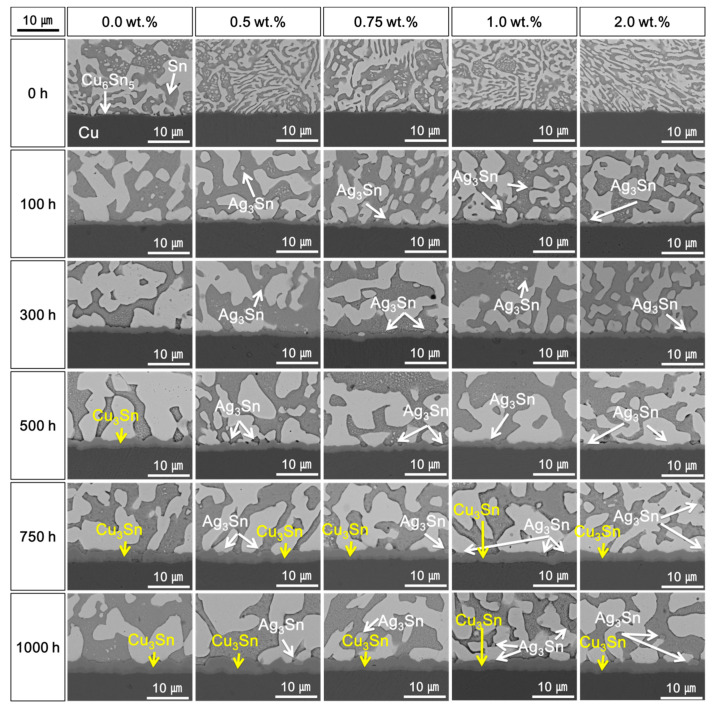
Cross-sectional scanning electron microscopy (SEM) micrographs of the Sn–58Bi Ag NP composite solder joints after high-temperature storage (HTS) tests at 115 °C.

Figure 4 also shows SEM micrographs of the cross-sectioned solder joints after 1000 h at 115 °C. In the samples without Ag NPs, Cu_3_Sn IMC was formed at the interface between the Cu_6_Sn_5_ IMC and Cu substrate after 500 h. With increasing Ag NP content, the IMC’s thickness decreased. Regardless of the temperatures for the HTS tests, the IMC thicknesses and the IMC growth decreased with increasing Ag NP content. It is assumed that the Ag NPs affected the refining of the Sn–Bi microstructure and formed Ag_3_Sn IMC. This means that the Ag_3_Sn IMCs could suppress grain coarsening and IMC growth. According to a previous report, the IMC shape at the interface of the solder joint can be improved to form a scalloped IMC more uniformly when the Ag_3_Sn nanoparticles are placed appropriately. However, the IMCs thickness can increase when excessive Ag_3_Sn is added [27].

**Figure 5:**
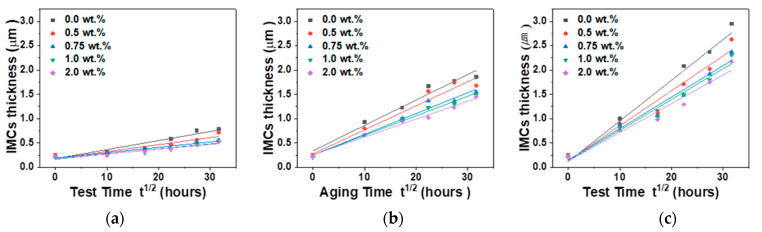
Total intermetallic compounds (IMCs) thickness as a function of test time at temperatures: (**a**) 85 °C (**b**) 100 °C, and (**c**) 115 °C.

The Cu–Sn IMC thicknesses in Sn–58Bi Ag NP composite solder joints as functions of the HTS test time and temperature are shown in Figure 5. The initial thickness of the Cu–Sn IMC did not change significantly with Ag NP content. The thicknesses of the IMCs increased with increasing test times and temperatures in the HTS tests. However, the growths and thicknesses of IMCs were smaller and slower in the solders containing Ag NPs compared to those without Ag NPs. We calculated activation energies to analyze the IMC growth rate of the Ag NP reinforced Sn–58Bi solder joints [28].

The relationship between IMC’s thickness and test time follows the Equation (1):

(1) $$w=kt^{n},$$

where *w* is the IMCs thickness, *k* the growth rate constant, *t* the test time, and *n* the time exponent. In addition, the activation energy required for IMC growth can be calculated in the solder joint by the following Arrhenius equation:

(2) $$k^{2}=k_{0}{}^{2}\exp\left(-\frac{Q}{RT}\right),$$

where *k^2^* is the square of the growth rate constant, *k*_0_ the frequency factor, *Q* the activation energy, *R* the gas constant (8.314 J/mol·K), and *T* the aging temperature in Kelvin (K).

(3) $$\ln\left(k^{2}\right)=\ln\left(k_{0}{}^{2}\right)-\frac{Q}{RT}$$

**Figure 6:**
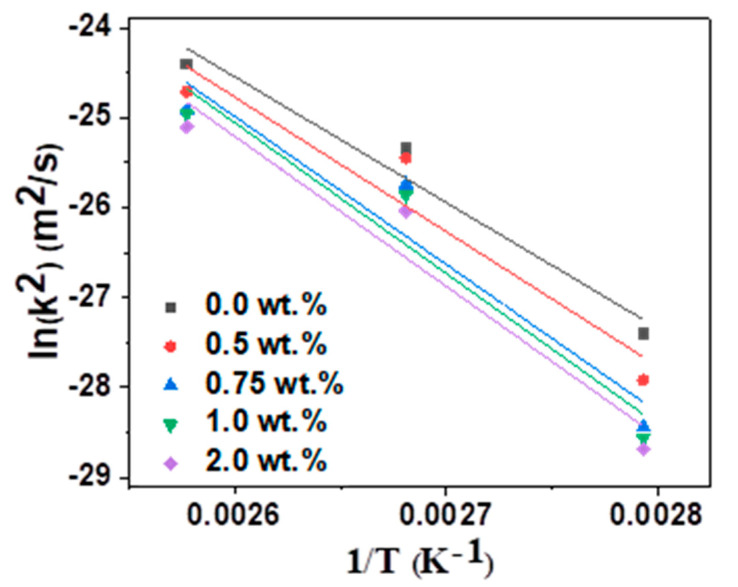
Arrhenius plots of intermetallic compound (IMC) growth rate as a function of Ag NP content of Sn–58Bi solder.

Equation (2) can also be converted to Equation (3) by taking the logarithms of both sides of Equation (2). Therefore, the Arrhenius plots were used to determine the activation energy for the growth of the IMCs during the HTS tests. These are shown in Figure 6. The activation energies were 115.6 kJ/mol for Sn–58Bi, 123.6 kJ/mol for Sn–58Bi–0.5Ag, 135.9 kJ/mol for Sn–58Bi–0.75Ag, 138.7 kJ/mol for Sn–58Bi–1.0Ag, and 138.0 kJ/mol for Sn–58Bi–2.0Ag. Therefore, the activation energy changed significantly with change in Ag NP content. It was assumed that IMC growth was inhibited by increased activation energies as the content of Ag NPs increased. According to previous studies, the activation energy between the Sn and 58Bi solder and the Cu substrate at 70–120 °C was about 127 kJ/mol [29,30]. In a previous study, adding 3 wt.% of Y_2_O_3_ to the Sn–58Bi solder increased the activation energy for Cu_6_Sn_5_ IMC growth by 14% when bonding on a Cu substrate [31]. However, in our study, the activation energy was increased by approximately 20% upon the addition of 2.0 wt.% Ag NP to the Sn–58Bi solder.

**Figure 7:**
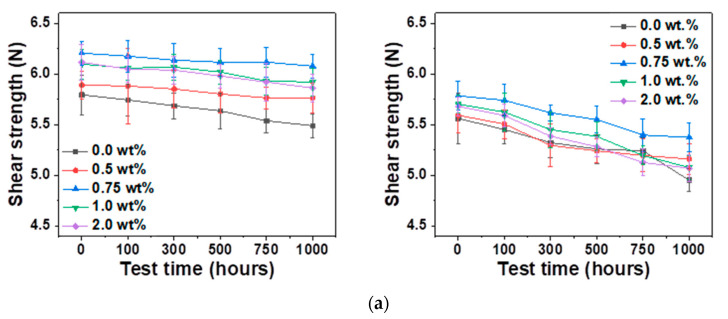

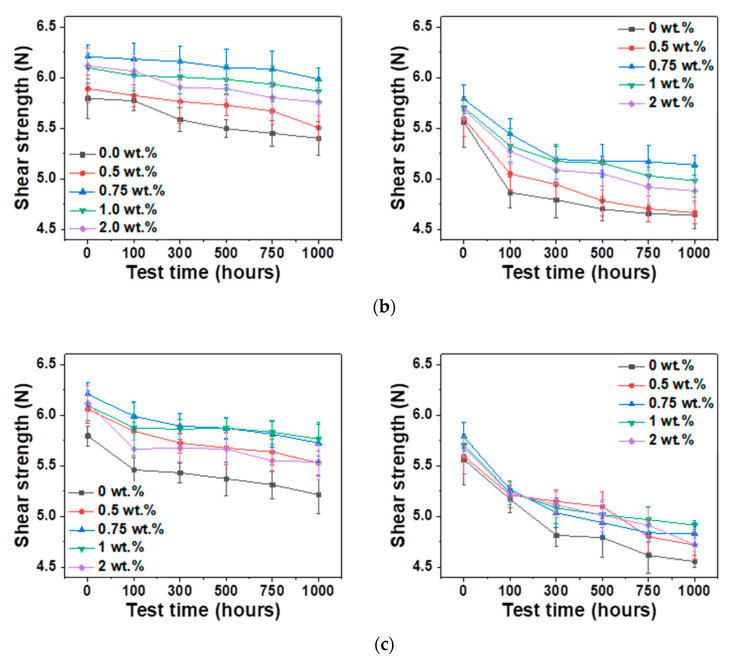
Shear strengths of Sn–58Bi composite solder joints with the test times and Ag NP contents after a high-temperature storage (HTS) tests at (**a**) 85 °C, (**b**) 100 °C, and (**c**) 115 °C.

Figure 7 shows the results of the shear tests at two shear speeds of 1.0 and 0.1 m/s. Results for different contents of Ag NP after HTS test at 85, 100, and 115 ℃ for 1000 h are presented. Overall, the Sn–58Bi composite solders with Ag NP showed higher shear strengths than the Sn–58Bi solder without Ag NP. Figure 7a shows the shear strength after the HTS test at 85 °C. The shear strength values of the Sn–58Bi composite solder with 0.75 wt.% Ag NP were 6.0 and 5.3 N at 1.0 and 0.1 m/s, respectively, which were higher than those of other Ag NP contents. The shear strength values after the HTS test at 100 °C were approximately 5.9 and 5.1 N for 0.75 wt.% Ag NP Sn–58Bi composite solder joint at shear speeds of 1.0 and 0.1 m/s, respectively (Figure 7b). However, after 1000 h at 115 °C, as shown in Figure 7c, the shear strength of the Sn–58Bi Ag NP composite solder joint with 1.0 wt.% Ag NP was the highest. Moreover, the shear strengths of the solder joints were decreased with increasing test times regardless of the contents of Ag NP and the temperature conditions of the HTS tests.

**Figure 8:**
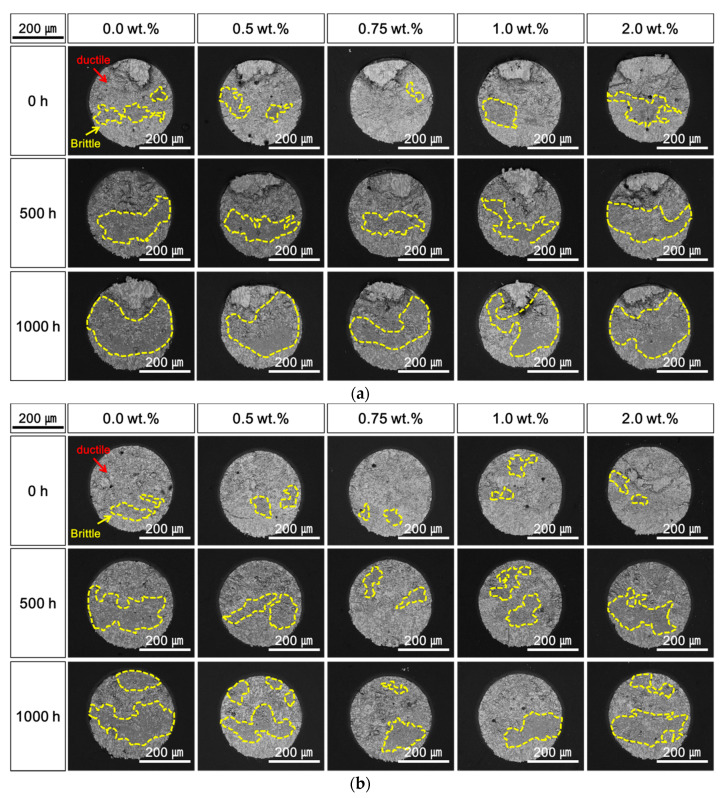
Top-view scanning electron microscopy (SEM) micrographs of fracture surfaces after a high-temperature storage (HTS) test at 85 °C: (**a**) 0.1 m/s and (**b**) 1.0 m/s.

**Figure 9:**
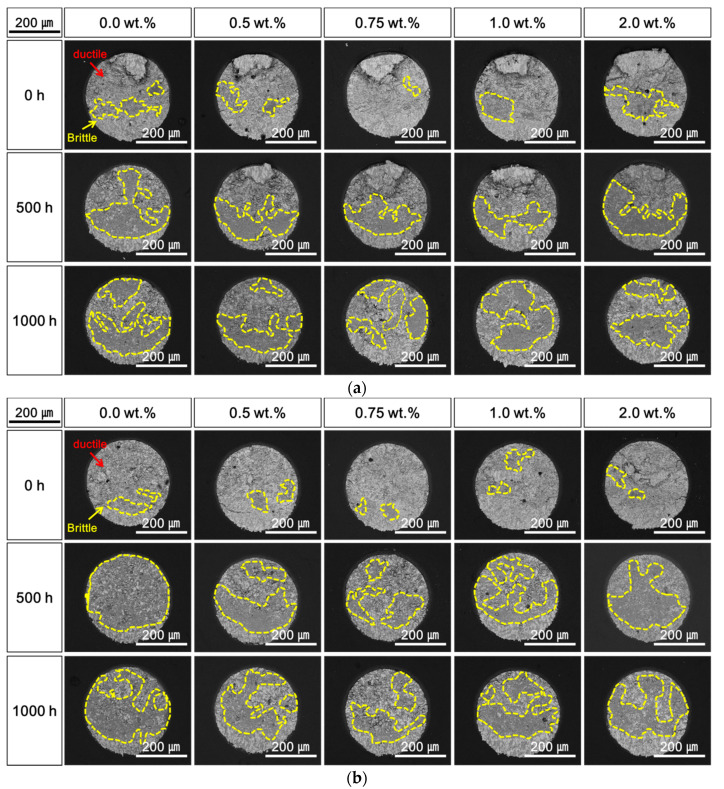
Top-view scanning electron microscopy (SEM) micrographs of fracture surfaces after a high-temperature storage (HTS) test at 100 °C: (**a**) 0.1 m/s and (**b**) 1.0 m/s.

**Figure 10:**
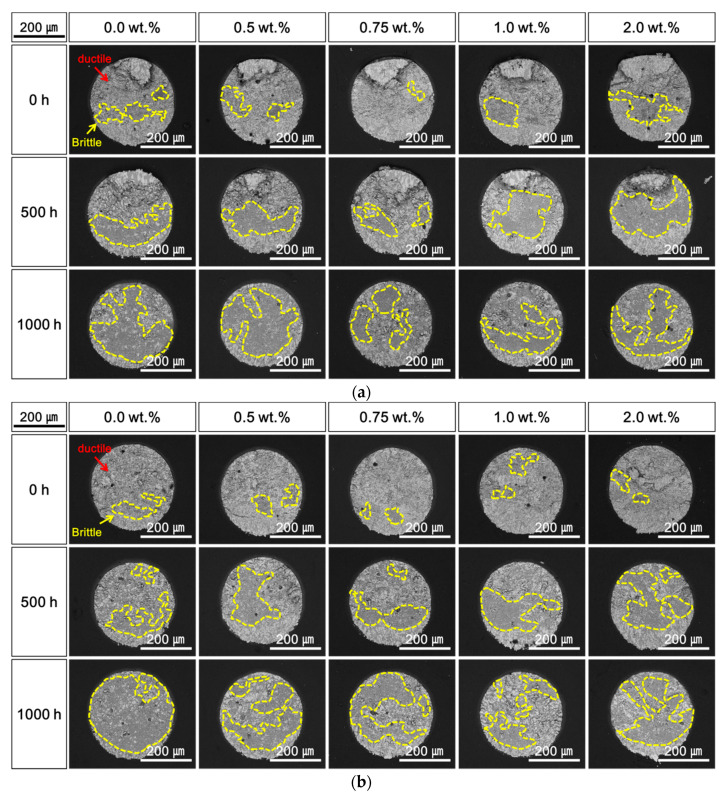
Top-view scanning electron microscopy (SEM) micrographs of fracture surfaces after a high-temperature storage (HTS) test at 115 °C: (**a**) 0.1 m/s and (**b**) 1.0 m/s.

Figure 8, Figure 9 and Figure 10 shows the top-view SEM micrographs of fracture surfaces after the shear tests performed during the HTS tests at 85, 100, and 115 °C. Both ductile fracture in the solder and brittle fracture at the Cu–Sn IMC layer occurred, and the two fracture modes were mixed under all conditions of the HTS tests and contents of Ag NP. At the initial stage after laser soldering, ductile fracture was dominant, and the addition of Ag NP did not significantly affect the fracture mode. The brittle fracture dimensions at the IMC surface increased with increasing HTS time and temperature, regardless of the Ag NP content. However, it was observed that the brittle fractures were suppressed when the Ag NPs were added to the Sn–58Bi solder. As shown in Figure 8, Figure 9 and Figure 10, the brittle fracture dimensions of solder joints with Ag NP were relatively small compared to those without Ag NP. In addition, the effects of the Ag NP increased as the lengths and temperatures of the HTS test were increased.

However, increases in the brittle fractures at the IMC layers were observed as the content of Ag NPs further increased up to 1.0 wt.%. The shear strengths of the solder joints also decreased when the Ag NP was 2.0 wt.%. As shown in Figure 2, Figure 3 and Figure 4, the Ag_3_Sn IMCs were formed in the solder and on the IMC layer at the interface of solder joints. This research assumed that coarsened Ag_3_Sn IMCs with increasing Ag NP content could adversely affect the shear strength and degrade the mechanical properties. In a previous study, the Ag_3_Sn was increased with increasing Ag NP content. However, the thickness of Cu–Sn IMCs such as Cu_6_Sn_5_ and Cu_3_Sn was thin, and the study reported that the addition of a specific amount of additives improves mechanical properties [14,15]. However, when the additive content exceeds a critical point, mechanical properties can be adversely affected. Therefore, our results were consistent with those of the previous study.

From our results in this research, a laser soldering method under low-temperature bonding was proposed, and its applicability to electronic packaging was confirmed. This research found the addition of Ag NPs suppresses IMC growth, which affects the increase in the activation energies. This research also observed that the addition of Ag NPs could improve the mechanical properties of solder joints in shear after HTS tests by decreasing brittle fracture due to inhibition of IMC growth [30,32]. However, this research also observed that the excessive addition of Ag NPs degrades the mechanical properties of the Sn–58Bi solder joint. Therefore, the addition of Ag NPs in the range from 0.5 to 1.0 wt.% using a laser process in the Sn–58Bi solder joint can improve the mechanical properties of the Sn–58Bi solder joint for low-temperature bonding of electronic packaging.

## 4. Conclusions

We investigated the interfacial reactions and mechanical properties of solder joints between Sn–58Bi solders, with and without the addition of 0 to 2.0 wt.% Ag NPs, and OSP-finished Cu, formed using a laser bonding method. Cu–Sn IMCs as Cu_6_Sn_5_ and Cu_3_Sn, were formed at the interface, even though the bonding times of the laser process were very short compared to the conventional reflow process. With increasing Ag NP content, the growth of Cu–Sn IMCs was suppressed, and the activation energies for the formation of Cu–Sn IMCs increased, regardless of the HTS temperature. It is inferred that Ag_3_Sn IMCs formed and that these IMCs could disturb the diffusion of Sn and Cu, which inhibited the growth of Cu–Sn IMCs. Both Cu_6_Sn_5_ and Cu_3_Sn IMCs were observed at the interface as the HTS temperatures and times were increased, while only Cu_6_Sn_5_ IMCs were formed after laser soldering. The addition of Ag NP to the Sn–58Bi solder joint improved the mechanical properties of the solder joints, as measured by shear tests, by significantly decreasing the brittle fracture due to reduced IMCs thickness. In all HTS tests at 1000 h, the Sn–58Bi composite solder with 0.75 wt.% Ag NP was 1 μm thinner than the Sn–58Bi solder, and the shear strength was about 7% higher. However, excessive addition of Ag NPs to 1.0 wt.% adversely affected the mechanical properties of the solder joint because the Ag_3_Sn IMCs coarsened at the interface and in the solder.

## Figures and Tables

**Table 1:** Compositions of solder pastes.

Specimens	wt.%	
	Ag NP	Sn–58Bi Powder
#1	0.0	100.0
#2	0.5	99.5
#3	0.75	99.25
#4	1.0	99.0
#5	2.0	98.0

## Data Availability

Data sharing is not applicable to this article.
